# Detection of *Bartonella alsatica* in European wild rabbit and their fleas (*Spilopsyllus cuniculi* and *Xenopsylla cunicularis*) in Spain

**DOI:** 10.1186/s13071-015-0664-1

**Published:** 2015-01-27

**Authors:** Francisco J Márquez

**Affiliations:** Departamento de Biología Animal, Biología Vegetal y Ecología. Facultad Ciencias Experimentales, Universidad de Jaén, Campus Las Lagunillas s/n, 23071 Jaén, Spain

**Keywords:** Andalusia, *Bartonella alsatica*, Fleas, ITS, European wild rabbit, *Odontopsyllus quirosi*, *Spilopsyllus cuniculi*, *Xenopsylla cunicularis*, Spain

## Abstract

**Background:**

*Bartonella alsatica* has been formerly isolated from the blood of wild European rabbit (*Oryctolagus cuniculus*) and identified as causative agent of human endocarditis and lymphadenitis. Fleas are known biological vectors for *Bartonella* sp. This report details the specific detection of *B. alsatica* in three flea species commonly associated with the European wild rabbit in Southern Iberian Peninsula (*Odontopsyllus quirosi*, *Spylopsyllus cuniculi* and *Xenopsylla cunicularis*).

**Methods:**

In the present study we have tested the presence of *Bartonella alsatica* in 26 European wild rabbit specimens and the fleas that they carrying at the moment of capture. Together to rabbits, captured from different localities of Andalusia (Jaen, Granada and Cordoba provinces), we evaluated three of fleas species that parasitize it usually using molecular techniques [PCR amplification and sequencing of intergenic transcribed spacer (ITS) 16S-23S rRNA].

**Results:**

Over a sample of 26 wild rabbits carrying fleas, positive PCR amplicons for *B. alsatica* were obtained from 10 rabbits. All positive flea pools for *B. alsatica* were collected from positive rabbits [33.33% (8/24 pools) of *S. cuniculi*, 33.33% (5/15 pools) of *X. cunicularis* and 0% (0/7 pools) of *O. quirosi*]. In three positive rabbits, a pool of *S. cuniculi* and two pools of *X. cunicularis* respectively were negative. After sequencing, only *B. alsatica* (Genbank accession AF312506) was found in the rabbits sampled as well as in *S. cuniculi* and *X. cunicularis* within the respective fleas.

**Conclusions:**

This research confirms the implication of two pulicidae flea species, *S. cuniculi* and *X. cunicularis* in the maintenance of infection by *B. alsatica* in wild rabbit populations throughout the year. The zoonotic character of this bartonellosis emphasizes the need to alert public health authorities and the veterinary community for the risk of infection.

## Background

Fleas carry and spread several bacterial diseases [1,2] of which *Bartonella spp.* are a facultative intracellular bacteria typically transmitted by blood-sucking arthropods, that cause characteristic host-restricted hemotropic infections in mammals [3]. *Bartonella alsatica* has been formerly isolated from the blood of wild European rabbit (*Oryctolagus cuniculus*) in Alsace Department, France [4]. In the previous years it has been also identified within a French study as causative agent of human endocarditis and lymphadenitis [5-7].

The blood feeding behavior of some arthropods plays a critical role in the transmission and maintenance of vector-borne pathogens in natural systems [8]. The application of molecular techniques for the detection of *Bartonella* foci has proven useful in the determination of vectorial capacity; furthermore molecular techniques used as a detection tool for fleas infected with *Bartonella* caught in nature is an essential tool for establishing a link between potential vectors and pathogens [9], whereas detection of DNA alone can be used as a preliminary step in determining which fleas are potential vectors and should be further studied.

Ectoparasites of European wild rabbit have provided insights into co-evolutionary processes between hosts [10-12]. The research governing the evolution of host-parasite interactions was discovered in the Iberian Peninsula and the South of France [13]. Five flea species were found on wild rabbits in Iberian Peninsula: *Spilopsyllus cuniculi* (Dale, 1878), *Xenopsylla cunicularis* Smit, 1957, *Odontopsillus quirosi* (Gil Collado, 1934), *Caenopsylla laptevi ibera* Beaucournu & Márquez, 1987, and *Echidnophaga iberica* Ribeiro *et al.*, 1994. *Spilopsyllus cuniculi* and *X. cunicularis* were the most prevalent and largely distributed fleas over rabbit populations [14]. The development and distribution of fleas, especially *S. cuniculi,* can be affected by the environment within the rabbit’s burrows [15,16]. Molecular detection of *B. alsatica* (taken from ear pinnae) has been recently reported with a prevalence of 17.2% over a large sample of wild rabbits in Southern areas of Iberian Peninsula [17].

## Methods

The presence of *Bartonella alsatica* in 26 European wild rabbit specimens were tested with all respective fleas that were found carried at the moment of capture. The rabbit specimens were captured from different localities of Andalusia (Jaen, Granada and Cordoba provinces) and evaluated. In these samples three flea species that typically parasitize rabbits has been found (*Spilopsyllus cuniculi, Xenopsylla cunicularis* and *Odontopsyllus quirosi*). Fleas collected from wild rabbits were suspended in a solution of 70% ethanol diluted with sterile water and stored in a refrigerator until ready for use. Flea species identification was performed using a regional taxonomic identification key [11]. Studied were 197 fleas distributed by host and species. Considered were 113 *S. cuniculi* (20 males, 93 females) in 24 pooled flea samples (average of 4.71 fleas per pool), 71 *X. cunicularis* (21 males, 50 females) distributed in 15 pools (average of 4.73 fleas per pool) and 13 *O. quirosi* (5 males, 8 females) distributed in 7 pools (average of 1.87 fleas per pool) (Table 1).

**Table 1:** **Flea pools composition**

***O. cuniculus***	**Σ** ***S. cuniculi***	***S. c.*** **results**	**Σ** ***X. unicularis***	***X. c*** **. results**	**Σ** ***O. quirosi***	***O. q.*** **results**
Pos.	6 (1 M, 5 F)	Pos.	7 (3 M, 4 F)	Pos.	1 (1 M, 0 F)	Neg.
Pos.	4 (0 M, 4 F)	Pos.	5 (2 M, 3 F)	Pos.	2 (1 M, 1 F)	Neg.
Pos.	5 (0 M, 5 F)	Pos.	6 (1 M, 5 F)	Pos.	-	-
Pos.	6 (2 M, 4 F)	Pos.	5 (3 M, 2 F)	Pos.	-	-
Pos.	5 (1 M, 4 F)	Pos.	4 (0 M, 4 F)	Neg.	-	-
Pos.	5 (2 M, 3 F)	Pos.	6 (1 M, 5 F)	Neg.	-	-
Pos.	3 (0 M, 3 F)	Pos.	-	-	-	-
Pos.	3 (0 M, 3 F)	Pos.	-	-	-	-
Pos.	5 (2 M, 3 F)	Neg.	-	-	-	-
Pos.	-	-	6 (2 M, 4 F)	Pos.	-	-
Neg.	5 (0 M, 5 F)	Neg.	4 (1 M, 3 F)	Neg.	1 (1 M, 0 F)	Neg.
Neg.	6 (2 M, 4 F)	Neg.	6 (2 M, 4 F)	Neg.	3 (1 M, 2 F)	Neg.
Neg.	6 (1 M, 5 F)	Neg.	5 (2 M, 3 F)	Neg.	1 (0 M, 1 F)	Neg.
Neg.	3 (0 M, 3 F)	Neg.	5 (0 M, 5 F)	Neg.	-	-
Neg.	6 (2 M, 4 F)	Neg.	3 (0 M, 3 F)	Neg.	-	-
Neg.	7 (2 M, 5 F)	Neg.	2 (0 M, 2 F)	Neg.	-	-
Neg.	5 (1 M, 4 F)	Neg.	3 (2 M, 1 F)	Neg.	-	-
Neg.	4 (0 M, 4 F)	Neg.	4 (2 M, 2 F)	Neg.	-	-
Neg.	3 (0 M, 3 F)	Neg.	-	-	3 (0 M, 3 F)	Neg.
Neg.	2 (0 M, 2 F)	Neg.	-	-	-	-
Neg.	4 (1 M, 3 F)	Neg.	-	-	-	-
Neg.	5 (0 M, 5 F)	Neg.	-	-	-	-
Neg.	6 (2 M, 4 F)	Neg.	-	-	-	-
Neg.	6 (1 M, 5 F)	Neg.	-	-	-	-
Neg.	3 (0 M, 3 F)	Neg.	-	-	-	-
Neg.	-	-	-	-	2 (1 M, 1 F)	Neg.
Positives = 10	113 (20 M, 93 F)	Positives = 33.33% (8/24 pools)	71 (21 M, 50 F)	Positives = 33.33% (5/15 pools)	13 (5 M, 8 F)	Positives = 0.00% (0/7 pools)

Pos. = positive for *B. alsatica*; Neg. = negative for *B. alsatica*. M = male; F = female; *O. cuniculus: Oryctolagus cuniculus*; *S. cuniculi: Spilopsyllus cuniculi*; *X. cunicularis: Xenopsylla cunicularis*; *O. quirosi: Odontopsyllus quirosi*.

DNA from spleen of wild European rabbits and its fleas was extracted using the Macherey-Nagel DNA tissue Kit (Düren, Germany), according to the manufacturer’s instructions. During DNA extraction negative controls consisting of sterile water were included for every 15 samples. DNA extracts were stored at −20°C until further processing. DNA from each pool of fleas was tested by PCR using the primers, URBarto1 (5′- CTT CGT TTC TCT TTC TTC A) [18] and Balsatrev1 (5′- CTT CTC TTC ACA ATT TCA TT) [17] for the first amplification round, and Balsatfor2 (5′ – CGT TTC TCT TTC TTC AGA TG) and Balsatrev2 (5′- TCA CAA TTT CAT TAG AAC AAG) [17], which amplify specifically a fragment of the 16S-23S rRNA intergenic spacer region (ITS) of the *B. alsatica*. As negative control, in addition to extraction blanks, DNA from five wild rabbits, five pools of *Ctenocephalides felis* and four pools of *Pulex irritans* fleas, all negative for *B. alsatica,* has been included [19]. To test the presence of other *Bartonella* species in the studied samples we used other primer system as previously described [19]. To avoid PCR contamination, sample preparation, reactions set-up, and PCR amplifications were carried out in separate laboratories, with different lab coats and gloves.

PCR amplifications were carried out in a MJ Mini Personal Thermal Cycler (Biorad, Hercules, CA, USA). Each first PCR mixture round consisted of the following: 8 μl of DNA, 20 pmol of each primer, 200 μM of dATP, dCTP, dTTP, dGTP, 2.0 mM MgCl_2_, 0.033U of DNA polymerase in 1x *Taq* buffer advanced (5 Prime GmbH, Hamburg, Germany), and sterile distilled water to a final volume of 50 μL. PCR cycles included an initial 90 seconds denaturation step at 96°C, followed by 25 and 30 cycles, for first and second amplification rounds, of denaturation at 94°C for 30 seconds, annealing at 50 and 52°C for 30 seconds, and extension at 68°C for 60 seconds. Amplification was completed by holding the reaction mixture at 68°C for 7 minutes to allow complete extension.

As flea internal DNA quality control and to help in the molecular identification of fleas species, PCR amplification of a 658 bp fragment of the mitochondrial cytochrome c oxidase subunit I (COI) gene was done with the same conditions expressed above and standard DNA barcoding primers LCO-1490 F 5′-GGT CAA CAA ATC ATA AAG ATA TTG G and HCO-2198R 5′- TAA ACT TCA GGG TGA CCA AAA AAT CA [20] using an annealing temperature of 46°C.

PCR products were resolved by electrophoresis in 1.0% SeaKem agarose (Cambrex, Rockland, ME, USA) in 1× buffer Bionic (Sigma, St. Louis, MO, USA) gels using a 100 bp ladder as molecular weight marker (Eurogentec, Seraing, Belgium). Products containing positive results were purified by using the Montage PCR kit (Millipore, Bedford, MA, USA) prior to sequencing. Positive PCR products were sequenced using PCR primers and the GenomeLab DTCS- Quick Start kit (Beckman Coulter) and a CEQ 2000XL capillary DNA sequencer (Beckman Coulter) according to the manufacturer’s instructions. The resulting ITS sequences were manually aligned and analyzed with Bioedit vers. 7.0.1. sequence analysis software [21] to obtain consensus sequences and to align and compare other *Bartonella alsatica* sequences found on GenBank, including previously sequenced and identified species of *Bartonella*, with homologous sequences obtained directly from rabbits tissues and fleas. Sequences were identified using the BLAST feature of GenBank (http://ncbi.nlm.nih.gov/blastn) [22]. PCR-derived flea COI gene sequences are deposited in GenBank under the accession numbers KF479234-KF479240 respectively for *S. cuniculi* (4 sequences), *X. cunicularis*, *Echidnophaga iberica*, and *O. quirosi*.

### Ethical approval

Ethical approval was given by the Universidad de Jaén Research Ethics Committee for the data collection. These approvals covered the extensive research protocols that were needed for sampling in the field and develop work at the molecular biology laboratory.

## Results and discussion

Eventually, all quality control assays performed on DNA of wild rabbit or fleas were positive. Overall, PCR amplicons positive for *B. alsatica* were obtained from 10 rabbits and 8/24 pools of *S. cuniculi* and 5/15 pools of *X. cunicularis* (Table 1). The 7 pools tested for *O. quirosi* were negative. All flea pools positive against *B. alsatica* were collected from positive rabbits. In three positive rabbits a pool of *S. cuniculi* and two pools of *X. cunicularis* respectively were negatives. After sequencing, only *Bartonella alsatica* was found in rabbit as well than in *S. cuniculi* and *X. cunicularis* fleas tested.

The sequences gathered for this study were further compared with other homologous sequences for *B. alsatica* previously accesed in GenBank (AF312506 and HM060955) and found to have a 100% amplicon homology with AF312506.

The molecular evidence depicts the persistence of *B. alsatica* in two rabbit flea species and shows that at least two of the five fleas evolutionary related with European rabbit, *Spilopsyllus cuniculi* and *Xenopsylla cunicularis* may have a role in the spread of *B. alsatica* among natural populations of wild rabbit, and possibly may be involved in the transmission of these bacteria to humans.

The study confirms the implication of at least two fleas in the transmission cycle of *B. alsatica* in European wild rabbit. In Andalusia, European wild rabbits have previously been found to be parasitized by 4 species of flea. Preliminary data indicates that at least 2 of these species (*S. cuniculi* and X*. cunicularis*) can contribute to maintain the transmission cycle of *B. alsatica* in nature. Two previous reports indicate molecular detection of *B. alsatica* from *S. cuniculi* fleas from a European wildcat (*Felis silvestris silvestris*) in Andalusia, Spain [19] and from wild rabbits from southern France [23] using PCR and sequencing. In rabbits and fleas studied, we found a DNA sequence homologous to AF312506, with a length of 1273 bases [24], whereas other authors [20] described a sequence of 576 bases in length (accession code HM060955) of *B. alsatica* infecting *S. cuniculi,* which overlap with AF312506 between bases 399 and 993. The main difference between both Genbank accessed sequences is the deletion in HM060955 of the track TCTTATGAATTTATTTATA between positions 696 to 714 of AF312506 (97% homology) in HM060955. We consider that the prevalence of such variant in rabbits is very low in South Spain (less than 1%) [17]. A phylogenetic tree considering phylogenetic relations among *B. alsatica* and other *Bartonella* species appears in [23].

Other fleas species tested from several species of rabbit main predators (red foxes, dogs, cats, wild cats or lynxes) of the same environments such *Pulex irritans*, *Ctenocephalides canis* and *Ct. felis* were negative for *B. alsatica* [19]. In this study only *S. cuniculi* from wild cats has been positive for *B. alsatica*, although in this case, it seems likely that these fleas come from rabbits predated by this feline.

In the Iberian Peninsula, the population dynamics of these two flea species show clear differentiated peaks of abundance. Adults of *S. cuniculi* were found largely parasitizing rabbits in spring, though *X. cunicularis* is more abundant on rabbits in summer [14,25], whereas the larvae of wild rabbit fleas shares the same rabbit burrows [26]. The reproductive cycle of *S. cuniculi* depend on the reproductive cycle of it host in relation with hormone availability [27,28]. Frequently adults of both species can be recovered from rabbits as well in several rabbit predators [19,29].

The overlapping and succession in time of these two fleas species as adult (or as larvae in the rabbit burrows) can explain the maintaining of *Bartonella alsatica* in rabbit population over the year. There is evidence to suggest *Ctenocephalides felis* faeces contains *Bartonella henselae* [30], and recently, it had been demonstrated that gut voids from the digestive tract of *Bartonella*-positive *Xenopsylla ramesis* contained *Bartonella* DNA [31]. On the other hand, the larvae of the flea *Parapulex chephrenis* include in its diet, faeces and voids from adult flea as well as other materials [32]. Ingestion of fomites by flea larvae could constitute a nontraditional vertical nontransovarial proper infection source for uninfected flea larvae and may contribute to maintain these vectorial cycles in natural conditions [33], contrary what occurs in the case of others pathogens transmitted by fleas [34].

## Conclusions

The data points towards the action of two pulicidae flea species, *S. cuniculi* and *X. cunicularis* (as adults or as larvae), in the maintenance of infection by *B. alsatica* in wild rabbit populations throughout the year [35,36].

More work is needed to confirm that those or other rabbit parasitizing flea species are involved in *B. alsatica* transmission as well as determining in such cases, if infection of flea can occur at larval stage (e.g. via ingestion of contaminated blood contained in flea faeces or in gut voids from non-necessarily conspecific adult flea) or if it is a consequence of adult flea feeding upon a bacteremic host.

Future research will be needed to study the epidemiology of *B. alsatica* and other *Bartonella* in Andalusia as well as their association with infections in European wild rabbit, its fleas and humans, furthermore to discern if *B. alsatica* could be responsible for human cases with unknown fever or febrile illness in Iberian Peninsula.
